# Editorial: Systems Modeling: Approaches and Applications

**DOI:** 10.3389/fmolb.2020.620464

**Published:** 2020-12-09

**Authors:** Alberto J. Martin, Ernesto Perez-Rueda, Daniel Garrido

**Affiliations:** ^1^Centro de Genómica y Bioinformática, Facultad de Ciencias, Universidad Mayor, Santiago, Chile; ^2^Instituto de Investigaciones en Matemáticas Aplicadas y en Sistemas, Universidad Nacional Autónoma de México, Unidad Académica de Yucatán, Mérida, Mexico; ^3^Departamento de Ingeniería Química y Bioprocesos, Pontificia Universidad Católica, Santiago, Chile

**Keywords:** systems modeling, biological networks, computational methods, network inference, network analysis

## Introduction

Systems Biology, a relatively recent discipline relies on computational modeling as one of its main tools. Ever appearing computational approaches allow us to raise new hypotheses that were unfeasible to test a few years ago. Given the broad range of applications of systems biology, we considered necessary to increase the coverage of tools and their applications in several areas, such as medicine, biotechnology and engineering.

The main goal of the Research Topic (Systems Modeling: Approaches and Applications) was to provide an overview covering both research articles and reviews. In this regard, the collection highlights the impact of computational tools and the usefulness of modeling to decipher the inner workings of biological systems.

Galán-Vásquez and Perez-Rueda evaluated co-expression networks for 17 bacterial organisms via weighted gene co-expression network analysis and clustered into modules of genes with similar expression patterns for each species, to determine relevant modules through a hypergeometric approach based on a set of transcription factors and enzymes for each genome.

Next, Cortés et al., constructed the regulatory and metabolic networks of the bacterium Acidithiobacillus thiooxidans, using an *in silico* semi-automatic genome scale approach. The authors provide an elegant identification of confident connections between both networks (V-shapes), identifying a sub-network of transcriptional factors (34 regulators) regulating genes (61 operons) encoding for proteins involved in biomining-related pathways. In contrast, pathways involved in iron homeostasis and oxidative stress damage are mainly regulated by unique primary regulators, conferring Licanantay an efficient, and specific metal resistance response.

In the third article, Khatami et al. make an excellent review describing the models to characterize Alzheimer's Disease. In this context, integrative models can be sorted in hypothetical models and data-driven models. The latter group split into two subgroups: (i) Models that use traditional statistical methods such as linear models, (ii) Models that take advantage of more advanced artificial intelligence approaches such as machine learning. The review highlights advancements of integrative modeling in the field of AD research.

Medina-Ortiz et al. explored an approach of unsupervised learning algorithms, and a new methodology designed to find optimum partitions within highly non-linear datasets that allow deconvoluting variables and improve performance metrics in supervised learning classification or regression models. These algorithms provide an excellent approach to generate predictive models for highly non-linear datasets; with not significant human input, which guarantees a higher usability in the biological, biomedical, and protein engineering community with no specific knowledge in the machine learning area.

Finally, Tsirvouli et al. show how a relatively large manually curated logical model can be efficiently enhanced further by including components highlighted by a multi-omics data analysis of data from Consensus Molecular Subtypes covering colorectal cancer; finding that the approach can benefit *in silico* experiments on cancer cell lines.

We believe as Editors of this topic, that the original aims have been fulfilled. We consider that the five articles (four original and one review), cover diverse descriptions and proposals to evaluate the modeling to understand the complexity of the biological systems. We must appreciate the works and authors for their excellent contributions that allow for inspiration for other professors in the field.

## Author Contributions

All authors listed have made a substantial, direct and intellectual contribution to the work, and approved it for publication.

## Conflict of Interest

The authors declare that the research was conducted in the absence of any commercial or financial relationships that could be construed as a potential conflict of interest.

